# Research Status and Prospect for Vibration, Noise and Temperature Rise-Based Effect of Food Transport Pumps on the Characteristics of Liquid Foods

**DOI:** 10.3389/fnut.2022.884835

**Published:** 2022-05-13

**Authors:** XiaoQi Jia, Songyu Li, Bo Li, Li Zhang, Qiangmin Ding, Panlong Gao, ZuChao Zhu

**Affiliations:** ^1^Key Laboratory of Fluid Transmission Technology of Zhejiang, Zhejiang Sci-Tech University, Hangzhou, China; ^2^Hangzhou Weiguang Electronic Co., Ltd., Hangzhou, China; ^3^Department of Application and Engineering, Zhejiang Economic & Trade Polytechnic, Hangzhou, China; ^4^Hefei General Machinery Research Institute, Hefei, China

**Keywords:** vibration, noise, temperature rise, food transport pump, liquid foods

## Abstract

In the field of food processing, the processing of liquid foods has always played an important role. Liquid foods have high requirements for the processing environment and equipment. As the core equipment in liquid foods processing, food transport pumps are widely used in liquid foods production, processing and transportation. Most liquid foods are non-Newtonian and vulnerable to vibration, noise, and temperature rise produced by rotary motions of food transport pumps in operation, which can finally affect foods safety. Therefore, this review summarizes the impact of mechanical vibration, noise, and temperature rise on liquid food products, with the aim of ensuring food safety while designing a cleaner, safer and more reliable food transport pumps in the future.

## Introduction

With the continuous development of economy and society, food safety is not only a major public health safety issue (1), but also a major issue related to people's survival and development and nutritional health (2–4). The impact of all links of food production on food safety cannot be ignored, especially in food processing and transportation (5–7). The processing of liquid foods such as peanut butter, beer, and milk makes enormous demands on delivering equipment. Due to the different molecular structures and physical parameters of various liquids, the impact of physical parameters of various equipment operation (mainly including vibration, noise and temperature rise) on liquid foods should be considered during the transportation of liquid foods (8–11). Food transport pumps accelerate the output of liquid food under pressure, thus realizing efficient and stable conveyance of liquid foods. It is the core delivering equipment in the production process of liquid foods. The most used two types of food transport pumps are vane pumps and positive displacement pumps (12–14). Vibration, noise and temperature rise caused by long-term operation are ubiquitous in the use of food transport pumps (15–19). When delivering liquid foods, pressure pulsation exists in the flow field due to the rotor–stator interaction between the vanes and volutes of the pump. Such pressure pulsations will result in the vibration and noise of the pump (20–23). In addition, local temperature rise of liquid foods in pump impeller and volute will occur during operation of food transport pumps, which will affect the safety of liquid foods (24, 25). Vibration, noise, and temperature rise of liquid foods are inevitable in the process of transfer. Extensive research efforts have been made to investigate the effect of such factors on the quality of liquid foods. This review focuses on the impact of liquid foods in the transfer process in terms of mechanical vibration, noise, and temperature rise.

## Effect of Vibration-induced Factors on Liquid Foods

Vibration in the process of transfer can negatively affect liquid foods (milk, liquor, yogurt, juice, etc.). For example, during yogurt fermentation, the vibration caused by large yogurt transfer pumps will spread to the fermenter (26), and the characteristic frequency of vibrations can affect pH and disturb protein network formation, which can lead to defects in yogurt texture (27). Körzendörfer et al. (28) tested the effect of vibration on the fermentation process of yogurt, and showed that mechanical vibration causes yogurt to produce large particles on the millimeter scale during stirring, while this particle formation is mainly induced by changes in yogurt pH (29). According to particle image velocimetry results, vibration forces the yogurt to undergo vertical back and forth movements, which leads to local protein breakage during aggregation and gelation, making the yogurt more susceptible to syneresis during storage (30). Richmond et al. (31) studied the stability of yogurt during simulated transport in different secondary packaging. Textural defects caused by vibration include whey and cracked or completely destroyed coagulum. In contrast, agitated yogurt might suffer from structural losses (such as hardness) and phase separation in the process of transfer (32, 33). In addition, the vibration can affect the concentration of aldehydes, especially at higher storage temperatures (34). Jaskula-Goiris et al. (10) have studied the beer production process, and found that vibration can lead to intensified collisions between fluid molecules, which can cause beer to undergo oxidative reactions and thus become turbid.

However, studies also have showed that mechanical vibration also positively affects liquid foods. Stoforos et al. (35) examined the effect of vibration on thermal mixing of liquid foods during cooling of several highly viscous foods, potato puree, banana puree, applesauce, and cheese sauce was investigated, and the results showed that thermal mixing of liquid foods was improved under low frequency lateral vibration. Low frequency lateral vibrations can homogenize the temperature distribution of liquid foods while also accelerating food cooling. Kim et al. (36) found that resonance vibration could alleviate the membrane fouling problem of whole milk during filtration process, and resonance vibration could more effectively alleviate the fouling phenomenon of milk. Salek et al. (37) concluded that mechanical vibration can convert mechanical energy into thermal energy and enhance the hardness, storage modulus, and viscosity of cheese sauce. Warmińska et al. (38) studied the effect of vertical vibration (10–60 Hz, 0.5–2 h) on raw milk, and found that vibration increases electrical conductivity, while also altering the heat and clotting behavior of chymosin. Czerniewicz et al. (39) revealed that vibration decreased the pH of raw milk while increasing the amount of free fatty acids (40).

## Effect of Noise-Induced Factors on Liquid Foods

Both noise and acoustic wave characteristics have a significant effect on food safety (41–43). Ultrasound has a more pronounced physical effect on milk and dairy products, and related studies have shown that ultrasound has a distinct effect on the degree of emulsification and overall homogenization of milk and dairy products (44, 45). According to this research phenomenon, related researchers made low-fat dairy products by using ultrasound for separating emulsion and removing the fat layer (46, 47). Ultrasound has also been used to enhance the milk curding ability (48). O'Sullivan et al. (49) found that Ultrasound has been found to reduce micelle size and hydrodynamic volume of sodium caseinate, whey and milk protein isolates. Shanmugam et al. (44) studied flaxseed oils/milk emulsion composition, and found that ultrasound treatment improved the gel properties, gel strength, and elasticity, while reducing the gelation time of emulsions. Gursoy et al. (50) showed that ultrasound can postpone the separation of serum from milk and increase the viscosity of milk. Chandrapala et al. (51) concluded that ultrasound could accelerate the dissolution of powder in milk and the release of individual casein micelles into solution. Sfakianakis et al. (52) found that ultrasonicated milk samples also showed an increase in gel stiffness, clotting strength, final storage modulus, cohesiveness, and water holding capacity.

Aadi (53) found that ultrasound could improve turbidity values, antioxidant capacity, free radical scavenging activity, ascorbic acid of liquid foods such as phenolics, flavonoids and flavonols. Abid (54) found that ultrasound could enhance the concentration values of inactivated polyphenolic compounds and sugars in enzymes (polyphenolase, peroxidase, and pectin methylesterase) and microbial communities. Ultrasound could also effectively reduce the number of microbes in juice (55). Jiang et al. (56) found that ultrasound could enhance the antioxidant activity of fliud foods. Tomadoni et al. (57) found that ultrasound could effectively reduce the number of yeasts and molds in strawberry and kiwifruit juices.

## Effect of Temperature Rise Induced Factors on Liquid Foods

For most foods, temperature rise implies a deterioration of food quality. Temperature significantly affects microbial reproduction and speeds up food spoilage under appropriate humidity and oxygen conditions (58). Generally, within a certain range of temperature, when the temperature of foods rises by 10°C under constant moisture conditions, the enzymatic and non-enzymatic chemical reaction rate will double, and the rate of food spoilage will increase by 4–6 times (59). The increase in temperature also damages the internal organizational structure of food, thus seriously worsening the quality. Excessive heat can also denature proteins in foods, disrupt vitamins especially vitamin C in watery foods, or change the properties due water loss and deform foods (60). Therefore, the temperature rise of food transport pumps should be strictly controlled during the operation of liquid foods (61). High-protein foods such as milk and soybean milk are highly sensitive to temperature and greatly affected by temperature in the process of production. During yogurt transfer, the temperature should be controlled at around 5°C to avoid spoilage (62, 63). Al-Attabi (64) found that the physical and chemical reactions in heat treatment resulted in changes in milk flavor, which is different from the flavor of raw milk. The temperature increase of milk results in Maillard reaction, lipid degradation and thermal denaturation of whey proteins and milk fat globule membranes (65). In addition, some by-products of Maillard reaction are harmful to human health and can cause allergic reactions when severe (66). Zhang et al. (67) found that the longer the heat treatment time, the higher the heating temperature of milk, and the more extensive the Maillard reaction, which resulted in various unacceptable odor components. Wu et al. (68) found that the yogurt fermentation temperature might degrade yogurt quality with the growth of microbes, and that culture temperature during production had a significant effect on the physical characteristics of the final product. Higher temperatures exacerbated yogurt whey separation (69), which would result in a weak protein network with coarser microstructures and reduce the viscosity and smoothness of yogurt (70). Yang et al. (71) studied the effect of different fermentation temperatures on the quality of yogurt and metabolites, found that temperature rise caused different degrees of negative effects on the nutritional, physical characteristics and flavor of yogurt. Finally, all the above mentioned papers outcomes are shown in Table 1.

**Table 1:** A summary table of all mentioned papers outcomes.

**Characteristics**	**Product**	**References**	**Outcomes**
Vibration	Yogurt	(28)	Produce large millimeter-sized particles
	Yogurt	(31)	Cause texture defects
	Beer	(10)	Oxidation of beer
	Mashed Potatoes et al.	(35)	Improve thermal mixing of foods
	Whole milk	(36)	Reduce milk scaling
	Cheese sauce	(37)	Increase hardness, storage modulus and viscosity
	Raw milk	(38)	Alter heat and rennet coagulation behavior
	Raw milk	(39)	Decrease pH and increase the amount of free fatty acid
Noise	Milk	(49)	Reduce micelle size and hydrodynamic volume of sodium caseinate, whey and milk protein isolate
	Flaxseed Oil/Milk	(44)	Improve gel properties, gel strength and elasticity
	Milk	(50)	Delay serum separation and increased viscosity
	Milk	(51)	Accelerate the dissolution of powders
	Milk	(52)	Increase gel hardness, coagulation strength, final storage modulus, cohesion and water holding capacity
	Phenols and flavonoids	(53)	Improve food turbidity value, antioxidant capacity, free radical scavenging activity, ascorbic acid
	Enzyme	(54)	Enhance concentration values of inactive polyphenolic compounds and sugars
	Mulberry juice	(56)	Enhance antioxidant activity
	Strawberry and kiwifruit Juice	(57)	Reduce yeast and mold counts
Temperature rise	Yogurt	(63)	accelerate spoilage
	Milk	(64)	Maillard reaction
	Milk	(67)	Produce bad odor components
	Yogurt	(68)	Reduce viscosity and smoothness
	Yogurt	(71)	Negative effects of nutrition, physical properties, and flavor

## Conclusion and Perspective

Food safety has been a hotspot and sticking point in research. This review summarized the effect of vibration, noise, and temperature rise in the operation of food transport pumps on the physical, chemical, and structural characteristics of liquid foods. However, machinery vibration and ultrasound are also used for improving the taste of liquid foods, but other hazardous materials will also come with temperature rise. In general, machinery vibration, noise, and temperature rise have both positive and negative effects on liquid foods. Therefore, further research should proceed. At the beginning of food transport pumps design, it is necessary to take full account of its impact on specific food, such as milk, yogurt, wine, fruit juice, etc., to develop a more adjustable multi-scene food transport pump, which can adjust the rotating speed, flow rate and blade structure according to different liquid foods. Such multi-functional food transport pumps are also the main research and development direction of food machinery in the future. It is also necessary to take into account the material characteristics of food transport pumps, and introduce new technologies and materials, such as carbon nanomaterials, coating technology, which can improve the food transport pumps' damping capacity, sound and vibration absorption capacity, and environmental friendliness. All of this technology will reduce vibration, reduce temperature rise and protect food more safely, when liquid foods are transferred by food transport pumps.

## Author Contributions

XJ: methodology and writing-review and editing. SL: formal analysis, data curation, visualization, and original draft. BL: supervision. QD and PG: polish the article. ZZ: conceptualization. All authors contributed to the article and approved the submitted version.

## Funding

This work was supported by the National Natural Science Foundation of China (Grant No. 51906221), the Key Research and Development Program of Zhejiang Province (Grant No. 2022C01148), and the Joint Fund of Zhejiang Natural Science Foundation (Grant No.LZY21E060002).

## Conflict of Interest

BL was employed by Hangzhou Weiguang Electronic Co., Ltd., Hangzhou, China. The remaining authors declare that the research was conducted in the absence of any commercial or financial relationships that could be construed as a potential conflict of interest.

## Publisher's Note

All claims expressed in this article are solely those of the authors and do not necessarily represent those of their affiliated organizations, or those of the publisher, the editors and the reviewers. Any product that may be evaluated in this article, or claim that may be made by its manufacturer, is not guaranteed or endorsed by the publisher.

## References

[1] Xu L Zheng Y Zhou C Pan D Geng F Cao J. Kinetic response of conformational variation of duck liver globular protein to ultrasonic stimulation and its impact on the binding behavior of n-alkenals. LWT. (2021) 150:111890. 10.1016/j.lwt.2021.111890

[2] Shi Y Zhou K Li S Zhou M Liu W. Heterogeneous graph attention network for food safety risk prediction. J Food Eng. (2022) 323:111005. 10.1016/j.jfoodeng.2022.111005

[3] Silva VL Sanjuan N. Opening up the Black Box: A Systematic Literature Review ofLife Cycle Assessment in Alternative Food Processing Technologies. J Food Eng. (2019) 250:33–45. 10.1016/j.jfoodeng.2019.01.010

[4] Chen X Voigt T. Implementation of the Manufacturing Execution System in the food and beverage industry—ScienceDirect. J Food Eng. (2020) 278:109932. 10.1016/j.jfoodeng.2020.10993211407551

[5] Silva VL Sereno AM José DASP. Food industry and processing technology: on time to harmonize technology and social drivers. Food Eng Rev. (2018) 10:1–13. 10.1007/s12393-017-9164-8

[6] Boye JI Arcand Y. Current trends in green technologies in food production and processing. Food Eng Rev. (2013) 5:1–17. 10.1007/s12393-012-9062-z

[7] Dagdelen C Aday MS. The effect of simulated vibration frequency on the physico-mechanical and physicochemical properties of peach during transportation. J LWT—Food Sci Technol. (2020) 137:110497. 10.1016/j.lwt.2020.110497

[8] Zachwieja J. Stress analysis of vibrating pipelines. AIP Conf Proc. (2017) 1822:020017. 10.1063/1.4977691

[9] Zeng L Jansson LG Borjesson A. Piping vibration and vibration damage prevention through screening of dynamic susceptibility. In: Proceedings of the 2017 25th International Conference on Nuclear Engineering. (2017). p. 2. 10.1115/ICONE25-66658

[10] Jaskula-Goiris B Causmaecker BD Rouck GD Aerts G Paternoster A Braet J. Influence of transport and storage conditions on beer quality and flavour stability. J Inst Brew. (2018) 125:60–8. 10.1002/jib.535

[11] Sun X Liu S Zhang X Tao Y Boczkaj G Yoon JY. Recent advances in hydrodynamic cavitation-based pretreatments of lignocellulosic biomass for valorization. Bioresour Technol. (2022) 345:126251. 10.1016/j.biortech.2021.126251 34728352

[12] Tamime AY Robinson RK. Tamime and Robinson's Yoghurt: Science and Technology. 3rd ed. Woodhead Publishing Limited (2007). p. 1–791. 10.1533/9781845692612

[13] Shigemitsu T Nakaishi E Maeda M Araki Y. Influence of blade number on performance and internal flow condition of centrifugal pump for low viscous liquid food. Int J Fluid Machin Syst. (2021) 14:132–41. 10.5293/IJFMS.2021.14.1.132

[14] Wang L. Energy efficiency technologies for sustainable food processing. Energy Efficiency. (2014) 7:791–810. 10.1007/s12053-014-9256-8

[15] Ca OY Dou Y Huang Y Cheng J. Study on vibration characteristics of fracturing piping in pump-starting and pump-stopping water hammer. J Fail Anal Preven. (2019) 19:1093–104. 10.1007/s11668-019-00699-7

[16] Vasil'ev VA Chegurko LE. Rotor vibration and liquid pressure pulsation in a centrifugal pump. Chem Petrol Eng. (1979) 15:334–6. 10.1007/BF01155700

[17] Zeng G Li Q Wu P Qian B Huang B Li S. Investigation of the impact of splitter blades on a low specific speed pump for fluid-induced vibration. J Mech Sci Technol. (2020) 34:2883–93. 10.1007/s12206-020-0620-7

[18] Wu J Zheng S Wang C Yu Z. Study on pipeline self-excited vibration using transient fluid-structure coupling method. Int J Adv Manuf Technol. (2020) 107:4055–68. 10.1007/s00170-020-04983-x

[19] Sun X Yang Z Wei X Tao Y Boczka G Yoon JY. Multi-objective optimization of the cavitation generation unit structure of an advanced rotational hydrodynamic cavitation reactor. Ultrason. Sonochem. (2021) 80:105771. 10.1016/j.ultsonch.2021.105771 34689065 PMC8551246

[20] Jia XQ Cui BL Zhu ZC Zhang YL. Experimental Investigation of Pressure Fluctuations on Inner Wall of a Centrifugal Pump. Int J Turbo Jet-Eng. (2017) 36:401–10. 10.1515/tjj-2016-0078

[21] Cui B Zhang Y Huang Y. Analysis of the pressure pulsation and vibration in a low-specific-speed centrifugal pump. J Fluids Eng. (2021) 143:021201. 10.1115/1.4048691

[22] Huang WX Alben S. Fluid–structure interactions with applications to biology. Acta Mech Sin. (2016) 32:977–9. 10.1007/s10409-016-0608-9

[23] Xuan X Wang M Zhang M Kaneti YV Xu X Sun X. Nanoarchitectonics of low-dimensional metal-organic frameworks toward photo/electrochemical CO2 reduction reactions. J CO2 Utill. (2022) 57:101883. 10.1016/j.jcou.2022.101883

[24] Yuan M Tan D. Quantitative analysis of the influence of eccentricity on the thermal characteristics of in-wheel motor. J Mech Sci Technol. (2022) 36:991–1002. 10.1007/s12206-022-0145-3

[25] Mercy A Umamaheswari B Latha K. Reduced-order thermal behavior of universal motor-driven domestic food mixers/grinders using AC and DC supplies. J Power Electron. (2021) 21:1322–32. 10.1007/s43236-021-00268-y

[26] Pt A Ak B Jh B Es A. Vibrations as a cause of texture defects during yogurt manufacturing–Formation of vibrations and their propagation in dairy production lines. J Food Eng. (2020) 293:110369. 10.1016/j.jfoodeng.2020.110369

[27] Walstra P Wouters J Geurts TJ. Dairy Science and Technology, Second Edition. (2006). 10.1201/9781420028010

[28] Koerzendoerfer A Temme P Noebel S Schluecker E Hinrichs J. Vibration-induced particle formation during yogurt fermentation—Industrial vibration measurements and development of an experimental setup. J Food Res Int. (2016) 85:44–50. 10.1016/j.foodres.2016.04.004 29544851

[29] Krzendrfer A Temme P Schlücker E Hinrichs J. Vibration-induced particle formation during yogurt fermentation—effect of frequency and amplitude. J Dairy Sci. (2018) 101:3866–77. 10.3168/jds.2017-13905 29477530

[30] Ak A Pt B Al B Jh A. Vibrations as a cause of texture defects during the acid-induced coagulation of milk—fluid dynamic effects and their impact on physical properties of stirred yogurt. J Food Eng. (2020) 292:110254. 10.1016/j.jfoodeng.2020.110254

[31] Richmond ML Harte BR Gray JI Stine CM. Physical damage of yogurt. The role of secondary packaging on stability of yogurt. J food protection. (1985) 48:482–6. 10.4315/0362-028X-48.6.482 30943587

[32] Anna L Xinxin W Ruikang C Shujun X. Modeling the effect of vibration on the quality of stirred yogurt during transportation. J Food Sci Biotechnol. (2020) 29:889–96. 10.1007/s10068-020-00741-7 32582451 PMC7297902

[33] Tamime AY Robinson RK. RobinsonYoghurt: Science and Technology (third ed.), Woodhead Publishing Limited, Cambridge, UK (2007).

[34] Paternoster A Jaskula-Goiris B Causmaecker BD Vanlanduit S Springael J Braet J. The interaction effect between vibrations and temperature simulating truck transport on the flavor stability of beer. J Science Food Agricult. (2019) 99:2165–74. 10.1002/jsfa.9409 30302771

[35] Stoforos GN Farkas BE Simunovic J. Thermal mixing via acoustic vibration during continuous flow cooling of viscous food products. J Food Bioprod Process. (2016) 100:551–9. 10.1016/j.fbp.2016.07.008

[36] Kim SH Min CS. Fouling reduction using the resonance vibration in membrane separation of whole milk. J Indus Eng Chem. (2019) 75:123–9. 10.1016/j.jiec.2019.03.011

[37] Salek RN Vašina M Lapčík L Cerníková M Lorencová E Li P. Evaluation of various emulsifying salts addition on selected properties of processed cheese sauce with the use of mechanical vibration damping and rheological methods. J LWT Food Sci Technol. (2019) 107:178–84. 10.1016/j.lwt.2019.03.022

[38] Warminska M Kruk A Brandt W. Effect of vibration frequency and exposure time on the technological usability of fresh milk. Polish J Food Nutr Sci. (2006) 15–56:247–51. Available online at: http://journal.pan.olsztyn.pl/pdf-98706-30519?filename=30519.pdf

[39] Czerniewicz M Kruk A Kielczewska K. Storage stability of raw milk subjected to vibration. Polish J Food Nutr Sci. (2006) 15–56:65–70. Available online at: http://journal.pan.olsztyn.pl/pdf-98675-30488?filename=STORAGE%20STABILITY%20OF%20RAW.pdf

[40] Kamath S Wulandewi A Deeth H. Relationship between surface tension, free fatty acid concentration and foaming properties of milk. J Food Res Int. (2008) 41:623–9. 10.1016/j.foodres.2008.03.014

[41] Mason TJ Peters D. Practical Sonochemistry: Power Ultrasound Uses and Applications (second ed.), Elsevier, London (2003). p. 1–155. 10.1533/9781782420620.1

[42] Mason TJ Chemat F Ashokkumar M. Power ultrasonics for food processing. In: Gallego-Juarez JA Graff KF editors. Power Ultrasonics: Applications of High Intensity Ultrasound. Cambridge: Woodhead Publishing (2015). p. 815–43. 10.1016/B978-1-78242-028-6.00027-2

[43] Tiwari BK Mason TJ. Ultrasound processing of liquid foods. Novel Thermal Non-Thermal Technol Liquid Foods. (2012) 6:135–65. 10.1016/B978-0-12-381470-8.00006-2

[44] Shanmugam A Ashokkumar M. Functional properties of ultrasonically generated flaxseed oil-dairy emulsions. Ultrason Sonochem. (2014) 21:1649–57. 10.1016/j.ultsonch.2014.03.020 24713146

[45] O'Sullivan J Beevers J Park M Greenwood R Norton I. Comparative assessment of the effect of ultrasound treatment on protein functionality pre-and post-emulsification. Colloids Surf A Physicochem Eng Aspects. (2015) 484:89–98. 10.1016/j.colsurfa.2015.07.065

[46] Leong T Juliano P Johansson L Mawson R McArthur S Manasseh R. Temperature effects on the ultrasonic separation of fat from natural whole milk. Ultrason Sonochem. (2014) 21:2092–8. 10.1016/j.ultsonch.2014.02.003 24613647

[47] Leong T Johansson L Juliano P Mawson R Mcarthur S Manasseh R. Design parameters for the separation of fat from natural whole milk in an ultrasonic litre-scale vessel. Ultrason Sonochem. (2014) 21:1289–98. 10.1016/j.ultsonch.2014.01.007 24485394

[48] Zhao L Zhang S Uluko H Liu L Lv J. Effect of ultrasound pretreatment on rennet-induced coagulation properties of goat's milk. Food Chem. (2014) 165:167–74. 10.1016/j.foodchem.2014.05.081 25038663

[49] O'Sullivan J Arellano M Pichot R Norton I. The effect of ultrasound treatment on the structural, physical and emulsifying properties of dairy proteins. Food Hydrocoll. (2014) 42:386–96. 10.1016/j.foodhyd.2014.05.011

[50] Gursoy O Yilmaz Y Gokce O Ertan K. Effect of ultrasound power on physicochemical and rheological properties of yoghurt drink produced with thermosonicated milk. Emir J Food Agricult. (2016) 28:235–41. 10.9755/ejfa.2015-09-719

[51] Chandrapala J Martin G Kentish SE Ashokkumar M. Dissolution and reconstitution of casein micelle containing dairy powders by high shear using ultrasonic and physical methods. Ultrason Sonochem. (2014) 21:1658–65. 10.1016/j.ultsonch.2014.04.006 24798226

[52] Sfakianakis P Tzia C. Flavor and sensory characteristics of yogurt derived from milk treated by high intensity ultrasound. Ho CT Mussinan C Shahidi F Contis ET editors. Nutrition, Functional and Sensory Properties of Foods. UK: RSC publishing (2013). p. 92–97. 10.1039/9781849737685-00092

[53] Aadil RM Zeng XA Han Z Sun DW. Effects of ultrasound treatments on quality of grapefruit juice. Food Chem. (2013) 141:3201–6. 10.1016/j.foodchem.2013.06.008 23871078

[54] Abid M Jabbar S Hu B Hashim MM Hu B Lei S. Thermosonication as a potential quality enhancement technique of apple juice. Ultrason Sonochem. (2014) 21:984–90. 10.1016/j.ultsonch.2013.12.003 24373787

[55] Abid M Jabbar S Wu T Hashim MM Hu B Lei S. Sonication enhances polyphenolic compounds, sugars, carotenoids and mineral elements of apple juice. Ultrason Sonochem. (2014) 21:93–7. 10.1016/j.ultsonch.2013.06.002 23835397

[56] Jiang B Mantri N Hu Y Lu J Jiang W Lu H. Evaluation of bioactive compounds of black mulberry juice after thermal, microwave, ultrasonic processing, and storage at different temperatures. Food Sci Technol Int. (2014) 21:92–399. 10.1177/1082013214539153 24917651

[57] Tomadoni B Cassani L Ponce A Moreira MR Agero MV. Optimization of ultrasound, vanillin and pomegranate extract treatment for shelf-stable unpasteurized strawberry juice. LWT Food Sci Technol. (2016) 72:475–84. 10.1016/j.lwt.2016.05.024

[58] Aviara NA Haque MA. Moisture dependence of thermal properties of sheanut kernel. J Food Eng. (2001) 47:109–13. 10.1016/S0260-8774(00)00105-9

[59] Sablani SS Kasapis S Rahman MS. Evaluating water activity and glass transition concepts for food stability. J Food Eng. (2007) 78:266–71. 10.1016/j.jfoodeng.2005.09.025

[60] Chowdhury S Roy R Mandal BK. A review on energy and exergy analysis of two-stage vapour compression refrigeration system. J Int J Air Conditioning Refrig. (2019) 27:1930001.1–9. 10.1142/S2010132519300015

[61] AlbayatiO AZ Kumar R Chauhan G. Forced air precooling studies of perishable food products. Int J Food Eng. (2007) 3:8. 10.2202/1556-3758.1119

[62] Igaa B Ah A Vg A. Lactose hydrolysis and protein fortification pose an increased risk for the formation of Maillard reaction products in UHT treated milk products. J Food Composition Anal. (2019) 84:103308. 10.1016/j.jfca.2019.103308

[63] Elliott AJ Deeth HC Datta N Amenu B. Heat-induced and other chemical changes in commercial UHT milks. J Dairy Res. (2005) 72:442. 10.1017/S002202990500138X 16223459

[64] Al-Attabi Z D'Arcy BR Deeth HC. Volatile sulfur compounds in pasteurised and UHT milk during storage. Dairy Sci Technol. (2014) 94:241–53. 10.1007/s13594-013-0157-y

[65] Lee AP Barbano DM Drake MA. The influence of ultra-pasteurization by indirect heating versus direct steam injection on skim and 2% fat milks. J Dairy Sci. (2017) 100:1688–701. 10.3168/jds.2016-11899 28088421

[66] Lee CH Chen KT Lin JA Chen YT Hsieh CW. Recent advances in processing technology to reduce 5-hydroxymethylfurfural in foods. J Trends Food Sci Technol. (2019) 93:271–80. 10.1016/j.tifs.2019.09.021

[67] Zhang Y Yi S Lu J Pang X Xu X Lv J. Effect of different heat treatments on the Maillard reaction products, volatile compounds and glycation level of milk. J Int Dairy J. (2021) 123:05182–105182. 10.1016/j.idairyj.2021.105182

[68] Wu S Li D Li S. J. Bhandari Bhesh Yang B. L.. Effects of incubation temperature, starter culture level and total solids content on the rheological properties of yogurt. Int J Food Eng. (2009) 5:1–17. 10.2202/1556-3758.1436

[69] Ceja B Rka B Eor B Tkh A Agja B Sbs B. Processing of high-protein yoghurt—a review. Int Dairy J. (2019) 88:42–59. 10.1016/j.idairyj.2018.08.002

[70] Rpl A Mjm A Cap A Ss B Jalds A Amg B. Physicochemical and microbial changes in yogurts produced under different pressure and temperature conditions–ScienceDirect. J LWT. (2019) 99:423–30. 10.1016/j.lwt.2018.09.074

[71] Yang S Yan D Zou Y Mu D Wu J. Fermentation temperature affects yogurt quality: a metabolomics study. J Food Biosci. (2021) 42:101104. 10.1016/j.fbio.2021.101104

